# The hippocampus is not a geometric module: processing environment geometry during reorientation

**DOI:** 10.3389/fnhum.2014.00596

**Published:** 2014-08-05

**Authors:** Jennifer E. Sutton, Nora S. Newcombe

**Affiliations:** ^1^Department of Psychology, Brescia University CollegeLondon, ON, Canada; ^2^Department of Psychology, Temple UniversityPhiladelphia, PA, USA

**Keywords:** hippocampus, spatial reorientation, allocentric representation, geometric module, spatial cognition

## Abstract

The hippocampus has long been known to play a role in allocentric spatial coding, but its specific involvement in *reorientation*, or the recalibration of a disrupted egocentric spatial representation using allocentric spatial information, has received less attention. Initially, the cognitive literature on reorientation focused on a “geometric module” sensitive to the shape formed by extended surfaces in the environment, and the neuroscience literature followed with proposals that particular MTL regions might be the seat of such a module. However, with behavioral evidence mounting that a modular cognitive architecture is unlikely, recent work has begun to directly address the issue of the neural underpinnings of reorientation. In this review, we describe the reorientation paradigm, initial proposals for the role of the MTL when people reorient, our recent work on the neural bases of reorientation, and finally, how this new information regarding neural mechanism helps to re-interpret and clarify the original behavioral reorientation data.

Consider a problem often faced by travelers to new cities: despite a carefully planned trip using a city map, a traveler emerges from a subway station with no clear sense of which direction to walk to visit a famous building. The internal sense of direction crucial for forming an *egocentric* reference frame, or knowledge of locations in the environment relative to one’s own position, has been disrupted by the underground subway ride. Instead, the traveler is left to rely exclusively on her *allocentric* knowledge of the city layout, or map-like knowledge of the positions of landmarks relative to each other. If they are available and if the traveler is knowledgeable, directional cues such as the sun’s position in the sky might offer clues also. The process that is necessary at this point is called *reorientation*, or the process of using allocentric knowledge to recalibrate egocentric knowledge.

The hippocampus has been believed for many years to play an integral role in allocentric spatial encoding via place cells in rats (O’Keefe and Nadel, 1978), and humans (Ekstrom et al., 2003). The nature of its involvement in resolving the egocentric—allocentric mismatch required by reorientation has only occasionally been addressed, although there is a rich behavioral literature examining the cognitive processing during reorientation. In this paper, we briefly review the behavioral work on reorientation and the extant hypotheses about the role of the hippocampus. Furthermore, building on some of our recent work, we offer a new interpretation of reorientation behavior that incorporates a new understanding of the role of the hippocampus.

## Behavioral investigations of reorientation and the geometric module

**Figure 1:**
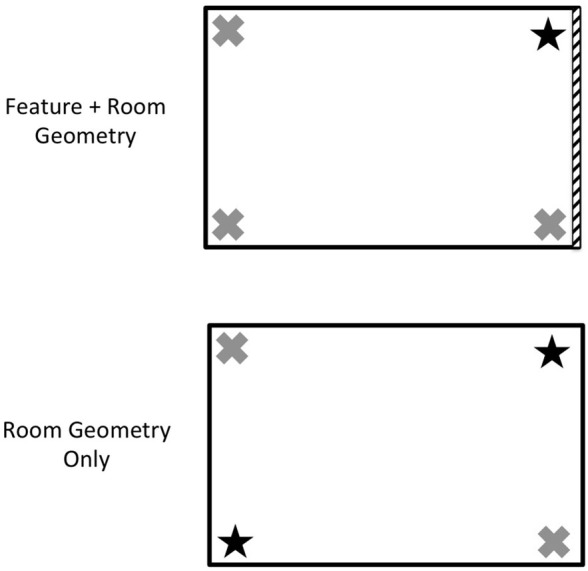
**Overhead views of typical rooms used to study reorientation**. On each trial, participants first see a target object hidden in one of the four corners, are then disoriented, and finally must point to the corner with the object. In the Feature + Room Geometry condition, three walls are identical and one is unique, so that participants may encode the target object (star) using room geometry (e.g., long wall on the left, short wall on the right), the position of the object relative to the unique wall, or both. In the Room Geometry Only condition, there is no feature to disambiguate the correct corner (upper right star) from its rotational equivalent (lower left star), so these two corners are chosen about equally.

Behavioral studies in the laboratory have addressed reorientation in a number of species and age groups that cross the traditional sub-disciplines of comparative, developmental, and cognitive psychology (reviewed by Cheng and Newcombe, 2005; Cheng et al., 2013). The paradigm, developed by Cheng (1986) for rats, has been implemented in a similar way across species and involves a rectangular room or arena and one or more distinct cues placed in the corners or on the walls (Figure 1). Subjects learn the location of a target object in a corner of the room in a learning phase, are then disoriented so that egocentric cues to the location are no longer valid, and subsequently try to locate the target object with only their allocentric representation of the space to guide them. Early studies of rats (Cheng, 1986) and young children (Hermer and Spelke, 1994, 1996) revealed a curious error pattern that indicated heavy reliance on information about the shape of the room or arena to locate the target, coupled with an apparent disregard for the information provided by the distinct cues within the room. Adult humans did not show this error pattern and successfully used both the room shape information, or its *geometry*, and the distinct cues within the room, which we will refer to as *features*, when finding the hidden object. These data suggested both species differences and a qualitative difference in how environment cues are used to reorient over development in humans (Hermer and Spelke, 1994; Ratliff and Newcombe, 2008).

Initially, the pattern of reliance on room shape when animals and young children reoriented was seen as an instance of a cognitive module dedicated to environment geometry, a cognitive mechanism that was both obligatory (i.e., always operating) and encapsulated (i.e., resistant to combination with other types of cue) (Cheng, 1986; Gallistel, 1990). Adults’ success was explained as a result of the use of language, unavailable to animals and young children, to combine the output of the geometric module with information from room features (Hermer-Vazquez et al., 1999). Animals’ and young children’s failure to use feature cues to disambiguate the information from room geometry was viewed as strong support for a modular mechanism. In addition, Gallistel also cast the proposal in an evolutionary framework with the idea that items in nature that give an environment its borders are usually stable characteristics, such as mountains and streams, whereas other, smaller, features such as the colors of leaves or flowers change regularly and are less effective spatial cues over the long term. Therefore, a cognitive module dedicated to processing the most reliable cues would be adaptive. The modular argument also fueled the nativist vs. empiricist debate, where some nativists proposed that the geometric module is innate and part of infants’ “core knowledge” (see Newcombe, 2002, for a review and critique).

However, as research using this technique (in the rest of this article, the “reorientation paradigm”) progressed, it became clear that the module proposal could not account for the diverse findings that emerged. For instance, manipulations of the room features, such as its size (Learmonth et al., 2002, 2008) and shape (Hupbach and Nadel, 2005) were shown to be critical in whether children used feature information along with room geometry. In addition, prior experience with the cues affected both children’s and adults’ reliance on different types of cue (Twyman et al., 2007; Ratliff and Newcombe, 2008). The necessity of language for successful cue combination has been called into question by demonstrations of successful geometry and feature use in multiple non-human species (chicks: Vallortigara et al., 1990; mountain chickadees: Gray et al., 2005; fish: Brown et al., 2007; pigeons: Kelly et al., 1998) and demonstrations of feature use when verbal processing is disrupted in adult humans (Hupbach et al., 2007; Ratliff and Newcombe, 2008).

With the existence of a dedicated geometric module for reorientation in doubt, a number of alternative accounts have been proposed that emphasize how cues are associated and which cues are used when (see Cheng et al., 2013 for a full review). For instance, Miller and Shettleworth (2007) proposed an account based on associative learning theory in which the features and geometry at a particular location compete for associative strength. The apparent dominance of geometric cues in some situations is a reflection of the associative strength of features enhancing the strength of the geometric cues. Another alternative is the adaptive combination model proposed by Newcombe and Huttenlocher (2006) and Newcombe and Ratliff (2007), in which feature and geometric cues are assigned different weights by the subject according to their perceived salience, reliability, and ease of encoding based on experience. The weights are then combined in a Bayesian manner with the greatest weight to the cues with the greatest perceived utility. Finally, a View Matching theory proposed by Stürzl et al. (2008) suggests that animals remember the visual panorama that accompanies a rewarded location, and subsequent searching is an attempt to decrease the discrepancy between the current view and the stored view. As noted by Cheng et al. (2013), each of these theories is consistent with a good portion of data from the reorientation paradigm, but none yet offers a complete explanation.

Given the large body of behavioral data from the reorientation paradigm accumulated at this point, continued progress arguably requires integrating behavioral findings with the neural mechanisms that underlie them. Until recently, studies using the reorientation paradigm and work on the neural bases of spatial learning have proceeded on parallel, rather than intersecting, research pathways. Understanding how the brain deals with these cues, whether these cues can even be differentiated at the neural level, and, if so, what commonalities can be seen in the activation during the reorientation paradigm and other tasks, can help in understanding reorientation specifically, and spatial behavior more generally.

## Initial hypotheses about the brain bases of performance in the reorientation paradigm

Initial hypotheses addressing the neural underpinnings of reorientation were based on fMRI studies that used very different tasks from the behavioral reorientation paradigm but were interpreted in terms of the modularity hypothesis. For instance, in one such non-reorientation task, Epstein and Kanwisher (1998) found extra-hippocampal activation in the medial temporal region that was associated with cues used in reorientation tasks. They showed subjects static images of rooms with objects, empty rooms, and objects in isolation and found that a posterior area of parahippocampal cortex, referred to as the parahippocampal place area (PPA), was activated similarly for all images with walls, regardless of whether objects were present. The same area also showed greater activation for walls alone vs. objects alone, and it was suggested that the PPA may be centrally involved in processing room geometry in the reorientation paradigm.

In another fMRI study that did not use the reorientation paradigm, Doeller et al. (2008) proposed that the hippocampus was the seat of a geometric module. Their study employed a virtual arena, modeled on the Morris water maze task used with rats, which consisted of a low wall as the arena’s boundary, distal cues outside the arena that provided orientation, and a traffic pylon within the arena that served as a landmark. The subjects’ task was to encode the locations of objects in the arena during an exploration period and to subsequently indicate the location of each object in the empty arena. On conflict tests, the landmark and the boundary indicated different locations for a target object. Greater hippocampal activation was observed when participants chose a target location consistent with information provided by the boundary vs. the landmark, and greater activation in dorsal striatum was found for landmark-consistent target locations. The apparent neural dissociation for the two types of spatial cue was interpreted as support for a cognitive dissociation, specifically, a geometric module. It was inferred, then, that the hippocampus is the primary supporter of a geometric module.

Differences between the Doeller et al. (2008) task and the reorientation task create problems for the hippocampus-as-geometric-module interpretation, however. Importantly, in the Doeller et al. (2008) study, distal orienting cues were located beyond and separate from the low boundary of the arena, the way cues may be placed on the walls of a laboratory room that houses a water maze or radial maze for rats. This arrangement makes it impossible to determine whether the observed hippocampal activation was associated with the boundary or with the distal cues beyond the boundary. That the hippocampus would show greater activation for distal cues in the Doeller et al. study makes sense given other fMRI data that confirm the importance of the hippocampus for processing similar cues in other tasks, such as a virtual radial maze task (Iaria et al., 2003). In the original reorientation studies of Cheng and others, however, the boundary of the room is the endpoint of the room, meaning feature cues can be placed *on* it but never beyond it. In order to address the neural underpinnings of reorientation, the effects of the orienting features and the boundary must be separable and performance with each compared, as in the original behavioral, “real life”, reorientation studies.

Our emphasis on the importance of the distal cues in the Doeller et al. (2008) task leads to a further question, however: if hippocampal activation on boundary trials is associated with using the distal cues to orient, shouldn’t these cues also have been used on landmark trials, resulting in similar hippocampal activation? We suspect that in the Doeller et al. task, the target localization process on landmark vs. boundary trials differs not only in use of the intended proximal cues (landmark, boundary) but also in use of the distal cues. Specifically, the process of replacing the target object based on memory (the “replace” phase) likely drew upon the distal cues to different degrees on landmark and boundary trials.

On boundary trials, the subject can determine the correct distance from the boundary for the target item without reference to the distal cues but *must* use the distal cues to find the particular point within the arena. Therefore, memory for the relationship of the distal cues to the target object is absolutely necessary to pinpoint the object’s location in the boundary condition.

On landmark trials, however, intra-arena cues can supply much of the needed information without as much reference to the orienting cues. For instance, one object is always placed adjacent to the landmark (see Doeller et al., 2008, Figure 1C); its location can be determined by remembering whether it was on the side of the landmark closer to the wall or the side toward the open arena. Since the landmark is never positioned dead center in the arena, distance to the wall is a reliable cue. For the object placed some distance away from the landmark, its direction from the landmark can be determined in one configuration by finding the point along the wall closest to the landmark, and for another by traveling toward the center of the arena from the landmark. With the other configurations, the distal cues seem more important for remembering the distance and direction of the object placed away from the landmark. All of these trials were averaged together, however, meaning that overall, the distal cues exerted less control over behavior on landmark trials.

In terms of the pattern of hippocampal activation over the two conditions, relatively greater reliance on distal cues should lead to greater hippocampal activation in the boundary condition than in the landmark condition. It should be noted that our interpretation of these data is not that the hippocampus is not involved in spatial coding using a boundary. Instead, we suggest it is impossible to conclude whether there is differential activation based on boundary or distal orienting cues. Despite this major issue, the pattern of neural activation demonstrated in the Doeller et al. study has been used as evidence that geometric reorientation is hippocampus-dependent and separable from feature use (Lee and Spelke, 2010).

## The hippocampus supports processing of both feature and geometry cues in reorientation

We have recently conducted studies that combine fMRI with a virtual reorientation task modeled closely on the behavioral task. This work reveals an important role for the hippocampus in reorienting, but one different in kind than previously thought. Sutton et al. (2010) had subjects actively move around a virtual room and encode the location of a traffic pylon in one of the corners in an encoding phase. Next, the screen was darkened for a few seconds and when the screen was re-illuminated, subjects were located in a randomly determined corner of the room and had to go to the center of the room, pick up a pylon, and place it in the location they had seen it in the encoding phase. On different trials, the room was either square with 1 red and 3 white walls (Feature room), rectangular with 1 red and 3 white walls (Feature + Geometry room) or rectangular with 4 white walls (Geometry only room). The Feature + Geometry and Geometry rooms replicated the conditions in the typical behavioral tests of reorientation, and the square Feature room provided a way to compare use of the Feature cue with informative geometry (Feature + Geometry) vs. the Feature in a room with uninformative geometry (Feature room). Of critical importance, the virtual task produced very similar response patterns and accuracy rates using the dependent measures typically employed in reorientation studies.

The pattern of brain activation revealed by pairwise comparisons of the room conditions in Sutton et al. (2010) revealed greater hippocampal activation when the room feature was present (Feature and Feature + Geometry rooms) vs. absent (the Geometry only room). This suggests that the relatively greater hippocampal activation shown by Doeller et al. (2008) for encoding a location relative to a boundary may have been driven by reliance on the orienting cues beyond the boundary, rather than the boundary itself. Therefore, these data can be viewed as consistent with Doeller et al. and Iaria et al. (2003), even though it is clear the hippocampus is not only associated with using a boundary for reorientation.

Relatively greater activation of the hippocampus when a feature cue is present should not be interpreted as indicating no role for the hippocampus in the processing of room geometry, however. To investigate medial temporal lobe processing of geometric cues more closely, Sutton et al. (2012) tested adults with another set of virtual rooms that presented different instantiations of a rectangular shape: pillars at the corners, complete walls, and a shaded rectangle flush with the ground. Adults produced the typical pattern of behavioral responses based on geometry (placements at the correct and rotationally correct corners) and there were no performance differences detectable between conditions. At the neural level, however, the pillars condition resulted in greater bilateral hippocampal activation when compared with the walls condition and the floor condition. This hippocampal involvement in encoding the rectangular shape of the 4-pillar configuration is consistent with other work showing a similar effect for learning a configuration of objects (Shelton and Gabrieli, 2002; Iaria et al., 2003; Bohbot et al., 2004).

Contrasts between the different geometry conditions revealed interesting extra-hippocampal activation differences in the medial temporal lobe, as well. Compared to the floor condition, the walls condition resulted in greater activation in bilateral areas of posterior parahippocampal cortex consistent with the PPA activation shown by Epstein and Kanwisher (1998). So, the spatial task was the same across conditions, but the presence of walls was associated with greater PPA activation. These data help clarify Epstein and Kanwisher’s original observation that the PPA is driven by images containing walls. When shape is held constant, the vertical and/or horizontal extent of the walls is associated with increased PPA activation.

In summary, instead of being the seat of a geometric module or, alternatively, associated only with the use of distal features, the hippocampus appears to be involved, but to varying degrees, for both feature and geometry processing in reorientation. Contrary to the geometric module idea, it seems that the two types of cue show commonalities at the neural level, rather than being completely different. At the cognitive level, it has already been suggested that in the natural world, extended surfaces that comprise geometric information and discrete feature/landmark cues serve a similar purpose for target localization and may be explained by the same cognitive mechanism (Sutton, 2009; Lew, 2011). Hippocampal involvement in processing both types of cue seems consistent with this idea and suggests new ways to interpret the original findings that drove the arguments for modularity.

## Viewing children’s reorientation behavior through the lens of hippocampal development

Initial findings that children up to about age 5 relied almost exclusively on the geometry of a room and ignored the features when reorienting were central to the geometric module proposal. In this way of thinking, these cues are seen as qualitatively different. As research on reorientation progressed, it became apparent that this pattern of behavior depended on certain conditions being met, such as a very small room where the walls and features were very close to the child. Even though a modular explanation is now doubtful, these data still require explanation: why is environment geometry so dominant, or alternatively, why are features so difficult, when children reorient in small rooms? Our new understanding of the role of the hippocampus when adults reorient offers insights into children’s reorientation behavior, as well.

Hippocampal volume increases dramatically between birth and age 2 but continues to develop, albeit more slowly, into childhood (Utsunomiya et al., 1999; Alvarado and Bachevalier, 2000; Seress, 2001; Gogtay et al., 2006). If we assume that conditions that are associated with relatively greater hippocampal activation in adults also demand more hippocampal resources during childhood, then hippocampal immaturity should be considered as an explanation for children’s reorientation behavior. Specifically, we would predict a match between conditions that are associated with greater hippocampal activation in adults and those in which children show performance deficits. Multiple findings from our studies with adults line up with children’s data along these lines. First, we found increased hippocampal activation when both a room feature and room geometry could be used to reorient, vs. just room geometry, using a virtual reality analogous to the original (Hermer and Spelke, 1994, 1996) task. Second, when room geometry was the only available cue, we saw a relative increase in hippocampal activation when that geometry was indicated by discrete objects at the corners rather than walls or a continuous floor. In line with this finding, children show performance decrements when faced with configurations of discrete objects for reorientation (Gouteux and Spelke, 2001; Lee and Spelke, 2008, 2011).

Beyond matching hippocampal activation in adults’ and children’s behavioral performance in this way, there should be evidence that other hippocampus-mediated cognitive processes are developing in early childhood, as well. Two cognitive processes associated with the hippocampus, place learning and memory binding, provide such examples. Additionally, they are directly relevant to reorientation performance.

Place learning refers to the ability to remember a location relative to multiple distal landmarks within an allocentric reference frame, and hippocampal activation is associated with place learning in adults (Iaria et al., 2003). Place learning begins to develop early, before age 2 (Newcombe et al., 1998; Sluzenski et al., 2004) and continues into school age (Learmonth and Newcombe, 2010). When young children have difficulty using room features in the reorientation task, it is when those features are very close to the child and not the distal cues the hippocampus is sensitive to in place learning. Generally, as the room gets larger in reorientation studies, young children are better at using the feature cues, suggesting that it is the close proximity and/or the size of the cues that is a problem in small rooms. In terms of hippocampal processing, the salience of the feature cues is reduced when they are presented at such a close distance. Therefore, an immature place learning system plus suboptimal cues could be the underlying cause for children’s inability to combine features and geometry to reorient. Immature place learning may also be implicated when young children have difficulty inferring the geometry of a space from objects placed in a rectangle.

Another candidate cognitive mechanism that may operate together with place learning is memory binding, or the process of connecting individual elements of a to-be-remembered situation in a rich memory representation (Cohen and Eichenbaum, 1993). This kind of relational memory has been shown to be hippocampus-dependent in adults within the shorter time frames required of the reorientation task (Olson et al., 2006; Hannula and Ranganath, 2008). The essence of the reorientation performance deficits of children is a failure to combine different sources of spatial information in memory, and indeed, binding is still developing in early childhood (Sluzenski et al., 2006; Lloyd et al., 2009). Thus, immature place learning and memory binding may work together to produce the kinds of deficits seen in young children.

Our discussion here focuses on the implications of hippocampal development, but it should be noted that the posterior parahippocampal gyrus has been shown in our studies to be particularly sensitive to rooms with walls. Little is known about how the function of the PPA develops in childhood, but differential development of this region relative to the hippocampus could be a factor in children’s performance. For instance, if the sensitivity to walls that we see in bilateral PPA in adults is present early in childhood, it may be that walls and, therefore, room geometry, are more salient to children than other types of cue. Given scant data on PPA development in children, however, this is an area for further research.

## Conclusion

The process of reorienting, or recalibrating disrupted egocentric spatial knowledge with allocentric spatial knowledge, is beginning to be understood at the neural level. The initial cognitive proposal of an impenetrable module that processed geometric information about the environment led to suggestions that the PPA or the hippocampus served as the site for such a module. Data now show both that cognitive modularity is doubtful and that the hippocampus is involved in processing multiple types of input to the allocentric representation people use when reorienting. Directly addressing the underlying neural mechanisms of reorientation not only clarifies the role of the hippocampus in this process but also suggests re-interpretation of some of the more puzzling reorientation behavioral data patterns. As pointed out by Cheng et al. (2013), the future of reorientation research likely involves its inclusion in more general, neurally-inspired models of spatial processing that make predictions about multiple spatial phenomena. For instance Sheynikhovich et al. (2009) have proposed a computational neural model of both reorientation and place learning in rats where views of the environment serve as input to a place learning system involving grid cells in entorhinal cortex and hippocampal place cells, while an action (taxon) system relays visual information to the dorsal striatum to guide movement via stimulus-response associations. Their model uses the place-learning features of the hippocampus to also explain performance in Cheng’s original reorientation task and ties together two behavioral literatures in animals that have traditionally been distinct. How and whether this type of model can account for more spatial phenomena and address multiple species remains to be seen, but this approach of addressing distinct behavioral tasks with the same model certainly holds promise for understanding the role of the hippocampus in reorientation and other spatial behaviors.

## Conflict of interest statement

The authors declare that the research was conducted in the absence of any commercial or financial relationships that could be construed as a potential conflict of interest.
